# Supplementary material for: Image-localized biopsy mapping of brain tumor heterogeneity: A single-center study protocol
Source: PLoS One. 2023 Dec 20;18(12):e0287767. doi: 10.1371/journal.pone.0287767 (PMC10732423; doi:10.1371/journal.pone.0287767)
Supplement: S1 Fig — For 1109/1130 flash-frozen samples, we have tracked the time from extraction to freezing. Switching from flash-freezing in pathology (N = 597) to inside the operating room (OR, N = 512) has allowed us to significantly reduce time between surgical extraction and freezing (linear mixed effects model with surgery ID as a nested random variable, p<0.0001). In doing so, we reach the goal of freezing biopsies in a 5-minute window (red dashed line) more consistently. (PDF) [file pone.0287767.s001.pdf]

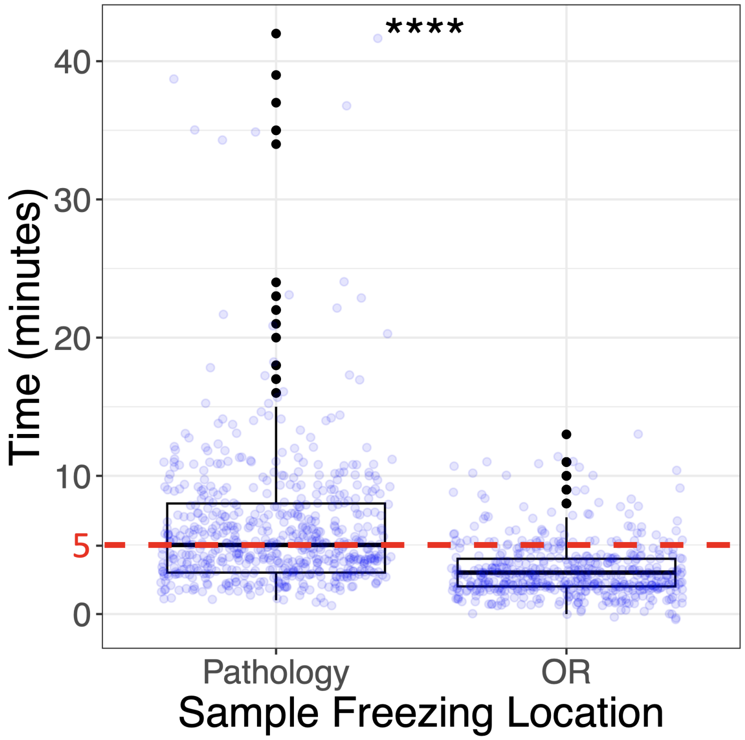

S1 Fig. Time taken to freeze biopsy samples. For 1109/1130 flash-frozen samples, we have tracked the time from extraction to freezing. Switching from flash-freezing in pathology (N=597) to inside the operating room (OR, N=512) has allowed us to significantly reduce time between surgical extraction and freezing (linear mixed effects model with surgery ID as a nested random variable, $p<0.0001$). In doing so, we reach the goal of freezing biopsies in a 5-minute window (red dashed line) more consistently.
