# Supplementary material for: Image-localized biopsy mapping of brain tumor heterogeneity: A single-center study protocol
Source: PLoS One. 2023 Dec 20;18(12):e0287767. doi: 10.1371/journal.pone.0287767 (PMC10732423; doi:10.1371/journal.pone.0287767)
Supplement: S2 Fig — Each sex consists predominantly of patients who identified as white, and patients who identified as not Hispanic/Latino. (PDF) [file pone.0287767.s002.pdf]

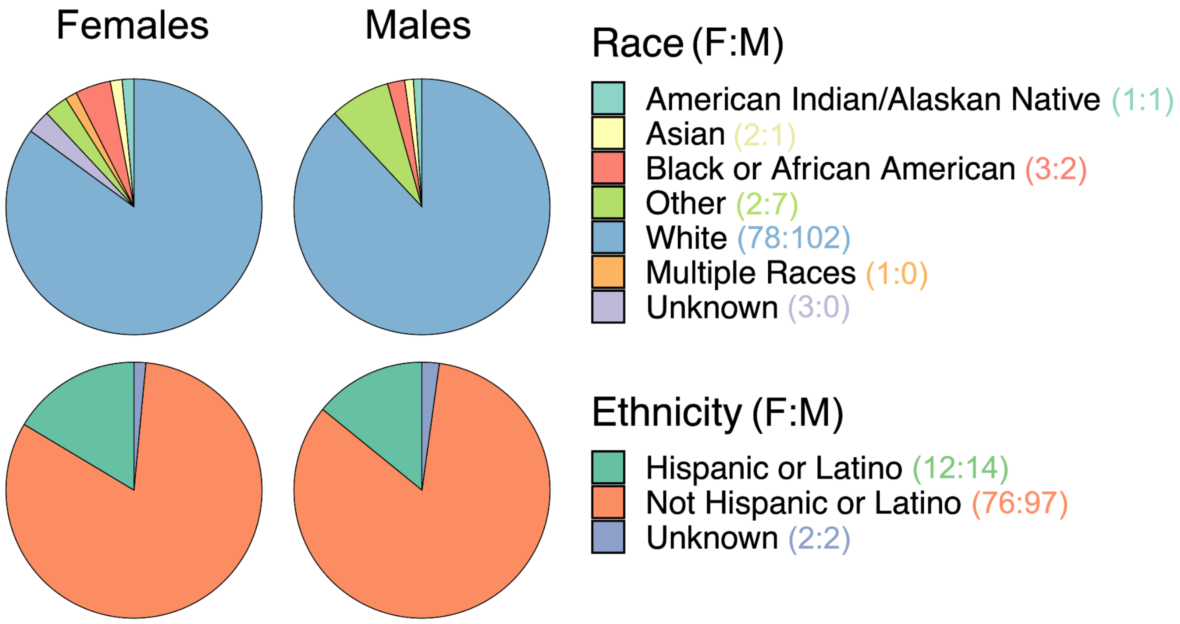

S2 Fig. Self-reported race and ethnicity broken down by self-reported sex of patients. Each sex consists predominantly of patients who identified as white, and patients who identified as not Hispanic/Latino.
