# Supplementary material for: Image-localized biopsy mapping of brain tumor heterogeneity: A single-center study protocol
Source: PLoS One. 2023 Dec 20;18(12):e0287767. doi: 10.1371/journal.pone.0287767 (PMC10732423; doi:10.1371/journal.pone.0287767)
Supplement: S3 Fig — The breakdown of patients approached and consented, as well as tumor type, grade and treatment status for patients who self-identified as female. *One case included in the recurrent grade III group underwent a grade transformation. (PDF) [file pone.0287767.s003.pdf]

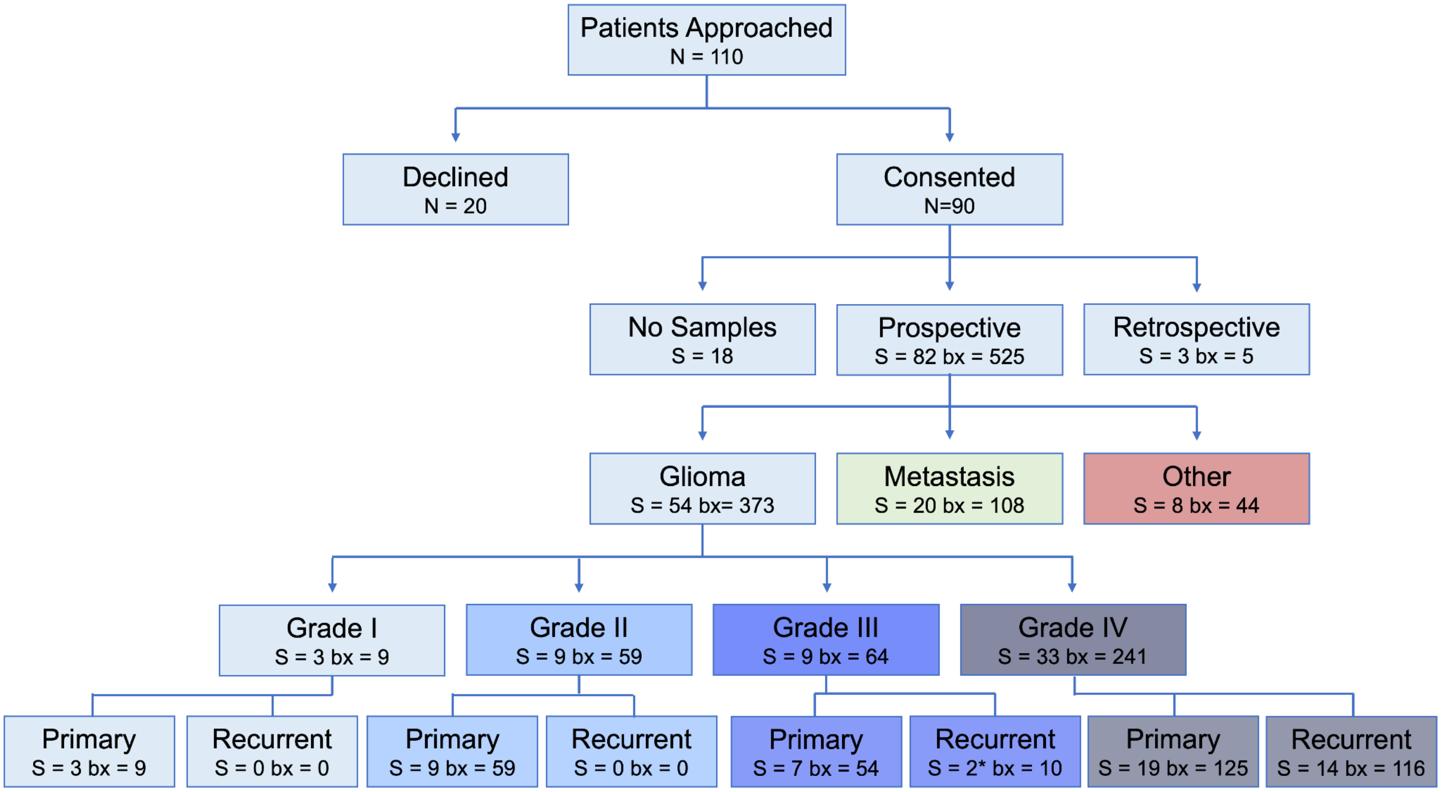

S3 Fig. Counts breakdown for female patients. The breakdown of patients approached and consented, as well as tumor type, grade and treatment status for patients who self-identified as female. *One case included in the recurrent grade III group underwent a grade transformation.
