# Supplementary material for: Image-localized biopsy mapping of brain tumor heterogeneity: A single-center study protocol
Source: PLoS One. 2023 Dec 20;18(12):e0287767. doi: 10.1371/journal.pone.0287767 (PMC10732423; doi:10.1371/journal.pone.0287767)
Supplement: S4 Fig — The breakdown of patients approached and consented, as well as tumor type, grade and treatment status for patients who self-identified as male. (PDF) [file pone.0287767.s004.pdf]

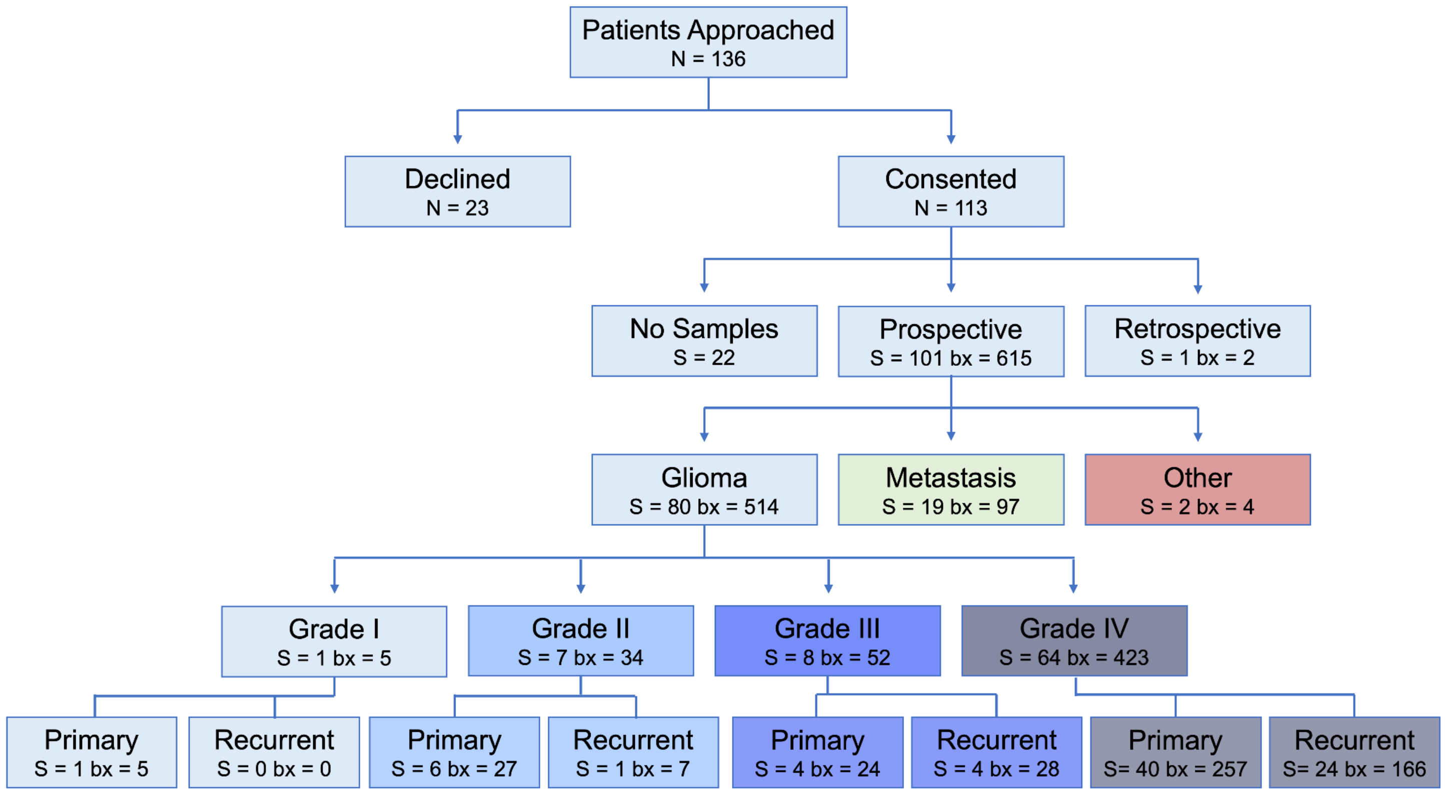

S4 Fig. Counts breakdown for male patients. The breakdown of patients approached and consented, as well as tumor type, grade and treatment status for patients who self-identified as male.
